# Benchmarking trial between France and Australia comparing management of primary rectal cancer beyond TME and locally recurrent rectal cancer (PelviCare Trial): rationale and design

**DOI:** 10.1186/s12885-016-2286-1

**Published:** 2016-04-04

**Authors:** Quentin Denost, Florence Saillour, Lindy Masya, Helene Maillou Martinaud, Stephanie Guillon, Marion Kret, Eric Rullier, Bruno Quintard, Michael Solomon

**Affiliations:** Department of Digestive Surgery, CHU Bordeaux, Saint André Hospital, Bordeaux, F-33075 France; Université Bordeaux Segalen, Bordeaux, F-33076 France; Unité Méthodes Evaluation en Santé, Centre Hospitalier Universitaire de Bordeaux, Bordeaux, France; Surgical Outcome Research Centre (SOuRCe), Royal Prince Alfred Hospital, University of Sydney, Sydney, New South Wales Australia; Unité de Soutien Méthodologique à la Recherche Clinique et Epidémiologique du CHU de Bordeaux (USMR), Université Bordeaux Segalen, Case 75, 146 rue Léo Saignat, 33076 Bordeaux, France; Laboratory of Psychology, Université Victor Segalen Bordeaux 2, Bordeaux, France; Department of Colorectal Surgery, Royal Prince Alfred Hospital, Sydney, NSW Australia; Service de Chirurgie Digestive, Hôpital Saint-André, 33075 Bordeaux, France

**Keywords:** Primary rectal cancer beyond total mesorectal excision plane (PRC-bTME), Locally recurrent rectal cancer (LRRC), Benchmarking study, Operative decision-making, Clinical pathway

## Abstract

**Background:**

Among patients with rectal cancer, 5–10 % have a primary rectal cancer beyond the total mesorectal excision plane (PRC-bTME) and 10 % recur locally following primary surgery (LRRC). In both cases, patients ‘care remains challenging with a significant worldwide variation in practice regarding overall management and criteria for operative intervention. These variations in practice can be explained by structural and organizational differences, as well as cultural dissimilarities. However, surgical resection of PRC-bTME and LRRC provides the best chance of long-term survival after complete resection (R0). With regards to the organization of the healthcare system and the operative criteria for these patients, France and Australia seem to be highly different. A benchmarking-type analysis between French and Australian clinical practice, with regards to the care and management of PRC-bTME and LRRC, would allow understanding of patients’ care and management structures as well as individual and collective mechanisms of operative decision-making in order to ensure equitable practice and improve survival for these patients.

**Methods/design:**

The current study is an international Benchmarking trial comparing two cohorts of 120 consecutive patients with non-metastatic PRC-bTME and LRRC. Patients with curative and palliative treatment intent are included. The study design has three main parts: (1) French and Australian cohorts including clinical, radiological and surgical data, quality of life (MOS SF36, FACT-C) and distress level (Distress thermometer) at the inclusion, 6 and 12 months; (2) experimental analyses consisting of a blinded inter-country reading of pelvic MRI to assess operatory decisions; (3) qualitative analyses based on MDT meeting observation, semi-structured interviews and focus groups of health professional attendees and conducted by a research psychologist in both countries using the same guides. The primary endpoint will be the clinical resection rate. Secondary end points will be concordance rate between French and Australian operative decisions based on the inter-country reading MRI, post-operative mortality and morbidity rates, oncological outcomes based on resection status and one-year overall and disease-free survival, patients’ quality of life and distress level. Qualitative analysis will compare obstacles and facilitators of operative decision-making between both countries.

**Discussion:**

Benchmarking can be defined as a comparison and learning process which will allow, in the context of PRC-bTME and LRRC, to understand and to share the whole process management of these patientsbetween Farnce and Australia.

**Trial registration:**

NCT02551471. (date of registration: 09/14/2015)

## Background

Over the last two decades, oncological outcomes for primary rectal cancer have improved due to refinements in neoadjuvant radiotherapy, chemotherapy and surgery. However, difficulties in resectability of the rectum come from its anatomical location and its close link to the pelvic skeleton, the lateral pelvic nerves and vessels, and the genitourinary system. These anatomical limits can affect the resectability of primary rectal cancer beyond the total mesorectal plane (PRC-bTME) and locally recurrent rectal cancer (LRRC). In fact, 5 to 10 % of cases of rectal cancer are bTME at the time of diagnosis with an extension to an adjacent organ (T4bNxM0) limiting their resectability [1, 2]. After radiochemotherapy, 30 % of these tumours will be resectable at the cost of extensive pelvic surgery and 10 % remain non-resectable [3]. Among patients operated for curative purposes (R0) for rectal cancer, 5 to 10 % will present LRRC within 5 years [4, 5] with a wide range of resectability rates between countries and institutions [6–10]. Nevertheless, surgery for curative purposes constitutes the “cornerstone” of the care and management of rectal cancer [11] for PRC-bTME [12–14] and LRRC [15–17], requiring multi-visceral resections associated with postoperative morbidity and mortality rates of 60 and 2 %, respectively [18]. The care and management of PRC-bTME and LRRC represents decisional conflict of the subjective and objective benefits and risks of the curative and palliative treatment options.

### The current state of knowledge

#### PRC-bTME

The 7th edition of the American Joint Committee on Cancer’s (AJCC) TNM - staging system (2010) [19] subdivided the T4 stage of rectal tumours in two sub-stages: stage T4a, in which the tumour has grown into the visceral peritoneum, and stage T4b in which the tumour is referred to as “fixed” or bTME and associated with a decrease in 5-year overall survival [20, 21]. In spite of a more pejorative prognosis of T4b tumours, extended multi-visceral surgical resection with a R0 margin allows 5-year survival identical to that of T3 or T4a tumours, at the cost of a higher post-operative morbidity rate [12, 13]. The resection rate of 35 % for PRC-bTME has been only reported in an Australian government report [22] regarding PRC-bTME and LRRC. No scientific publication has reported this to our knowledge.

#### LRRC

The rate of pelvic recurrence after rectal surgery for curative purposes has vastly diminished since the utilisation of pre-operatory radiotherapy [23] and the development of total mesorectal excision (TME) [11], varying from 5 to 10 % at 5 years [4, 5]. In half of the cases, recurrence is isolated in the pelvis. The resection rate for curative purposes (R0) of these pelvic recurrences varies in the literature from 22 to 74 % [24] and in a third of cases, excision is extended to adjoining organs (sacrum, uterus, vagina, prostate, bladder) [6] allowing to achieve a median survival from 19 to 31 months [6–10] and a 5-years overall survival of 40 % [25]. In the absence of surgical excision, the survival rate is lower than 4 % at 5 years with a median survival rate of 3.5 at 13 months [17]. The utilisation of radiotherapy or chemotherapy on their own only allows an improvement of the symptoms, without modifying survival. Several classifications aiming to predict resectability of this recurrence have been published taking into account: the symptoms [6], the site and degree of fixation [6, 26], the depth of colorectal-wall invasion and adjacent organs [27], the anatomical region of the pelvis invaded by the recurrence [28] and the dissection plans defined by the MRI after neoadjuvant treatment [29]. The resection rate of 42 % for LRRC has been only reported in an Australian government report [22] regarding PRC-bTME and LRRC. No scientific publication has reported this to our knowledge.

### Hypotheses of the research

This research project rests on the comparison between two countries that appear to contrast with regards to the care management of PRC-bTME and LRRC, France and Australia. Regarding its healthcare system for patients with PRC-bTME and LRRC, Australia equipped itself with a veritable policy of centralisation and clinical pathway, appearing as a national and international referral country in this surgical field [22]. On the other hand, France and other European countries [30] do not have this clinical pathway policy for patients with PRC-bTME and LRRC which may have resulted in high variability and heterogeneity of management for these patients.

The main hypotheses of research are that these differences rest on individual and collective representation of disease, organisations, structures, clinical pathway and care management. The understanding of this whole process will allow an optimisation of surgical indications and an improvement in patients’ survival. Benchmarking of clinical practices is the process of a structured comparison and the sharing of good practices of clinical care; it is based on a quality of care assessment and allows for continuous improvement of this quality of care.

In this context, a benchmarking-type analysis of surgical indications between France and Australia, but also the differences between organisations, care management systems for patients as well as in individual and collective mechanisms (representations, reasons, patterns) linked to surgical decision-making will allow a better understanding of these differences in practice and, over time, their homogenisation in order to improve care in both systems.

## Aims

The main objective of our study is to evaluate and compare, in a prospective benchmarking study, the operative-decision making and practice for PRC-bTME and LRRC in France and Australia.

Secondary objectives include assessment of concordance of operative-decisions made in MDT meetings, quality of surgery, post-operatory morbidity and mortality, disease-free survival, and overall survival, the quality of life and distress level of patients, the individual and collective mechanisms of decision-making and the structural and organisational contexts of care management between France and Australia.

## Methods and design

### Ethics statement

This non-interventional study has been approved by French and Australian national Institutional Review Boards: the Regional Comity of Patients Protection of South-West III (France), N°DC2015/22; and by the Sydney Local Health District Ethics Committee (Australia): HREC/15/RPAH/83&X15-0056; HREC/15/RPAH/123&X15-0091 and HREC/15/RPAH/208 &X15-0149. This study is supported by a grant from the French Ministry of Health (PREPS-14-0070). The institutional promoter is the Bordeaux University Hospital (DRCI: Direction de la Recherche Clinique et de l’Innovation).

### Consent statement

Before inclusion into this prospective study, all patients will sign consent for participation. As this trial is a non-interventional study, this consent will inform the patient about details regarding the study protocol, eligible criteria and objectives of the research.

### Population and design

The study protocol was conceived following an international benchmarking-type pilot study, allowing for the most rigorous assessment of the whole care management processes for patients with PRC-bTME and LRRC in France and Australia. Patients are included from 10 French and 2 Australian tertiary colorectal centres (see list of participating centers in the Acknowledgments section). Approval from all participating centers has been obtained. The study design includes three parts:

1. French and Australian national cohorts
  All patients must fulfil the following criteria: PRC-bTME or LRRC without distant metastases for curative, i.e. surgical resection, or palliative, i.e. no surgical resection, treatment intent. The complete inclusion and exclusion criteria are given in Table 1. This is a non-interventional study, no supplementary examinations to those performed in the framework of patient care management will be carried out for the inclusion of patients. According to the guidelines established by a consensus of international experts [1], before inclusion, the patients should have a pelvic MRI and a thoracoabdominal-pelvic scan. Patients should be included after MDT meeting discussion, irrespective of a curative or palliative treatment decision. Patients not included in the study, due to exclusion criteria, will be captured for calculation of incidence.

**Table 1:** Inclusion and exclusion criteria

Inclusion criteria	Exclusion criteria
• Primary rectal cancer (ymrT4bNxM0) or Local rectal recurrence cancer without distant metastases after partial or total mesorectal excision • Patient’s health condition which enable surgical • procedure and/or radiotherapy and/or chemotherapy • Age ≥18 years old • Oral agreement after reading information letter • Patient who benefits by medicare system	• Primary rectal cancer status lower than ymrT4b • Primary rectal cancer or Local recurrence rectal cancer with distant metastases • Patient’s health condition which don’t enable surgical procedure and/or radiotherapy and/or chemotherapy • Pregnancy or breast feeding period • Patient into the care of a guardian • Patient under the protection of the Court • Inability to give oral agreement due to bad comprehension capacity • Inability to be followed by medical team for geographic, social or psychologic reasons
  The data collected, in France as well as in Australia through a Clinical Report Form (CRF), will include clinical data, radiological data, (neo) adjuvant therapies, surgical and pathological data, quality of life and distress level. At inclusion, 6 and 12 months, the evaluation of quality of life and distress level will be collected using validated self-reported questionnaires, the MOS SF36 [31], FACT-C [32] and the Distress Thermometer [33], respectively.
  Patients will be followed in clinics according to he habits of each department. Usual follow-up is composed of clinical exam, CEA analysis, CT-scan or Chest Radiography with abdominal ultra-sound every 3–4months during the first three years and every 6 months for the last two years.
2. Blinded inter-country reading of pelvic MRI
  This experiment will consist of an inter-country reading of patients’ pelvic MRIs, “blind” to the other country’s decision. All centres will use the same MRI protocol with an uniform report. The MRI shared will be the one based on which the curative (surgery) or palliative care management decision (no surgery) the treatment decision will be made, that is to say, the MRI done after possible neoadjuvant treatment. Pelvic MRIs will be transferred after anonymization (Dicom Cleaner software®). In the case of medical contraindication to undergo pelvic MRI, the appropriate diagnostic scan will be used to assess the care-decision concordance between both countries.
3. Qualitative analysis
  Qualitative data will be collected by a research psychologist, based on MDT meeting observation, semi-structured interviews and focus groups of practitioners (surgeon, oncologist and radiologist) involved with the therapeutic decisions of patients with PRC-bTME and LRRC. The procedure of qualitative investigation will be carried out in an identical manner in France and Australia and will rest on the following methodological plan:
  MDT meeting observation (3 per centre) with “real” patient cases and “theoretical” patient cases (blinded pelvic MRI re-reading). This will be made in reference to the recommendations decreed by Mucchielli [34] and Norimatsu & Pigem [35]: a researcher in psychology will attend MDT meeting (“real” and “theoretical”) with the help of an observation guide previously established.
  Semi-structured exploratory interviews and focus group will be conducted with MDT health professional attendees (oncologists, radiologists and surgeons) to identify care management systems for PRC-bTME and LRRC patients, explore social representations that direct the formulation of a therapeutic decisions and identify cultural, medical and personal factors. Both semi-structured interviews and focus groups will be carried out using an interview guide with open questions and in the broadest sense possible in order to avoid any phenomena of induction [36]. Techniques such as word association, reminders and reformulations will be used during the interviews and focus groups.

**Fig. 1:**
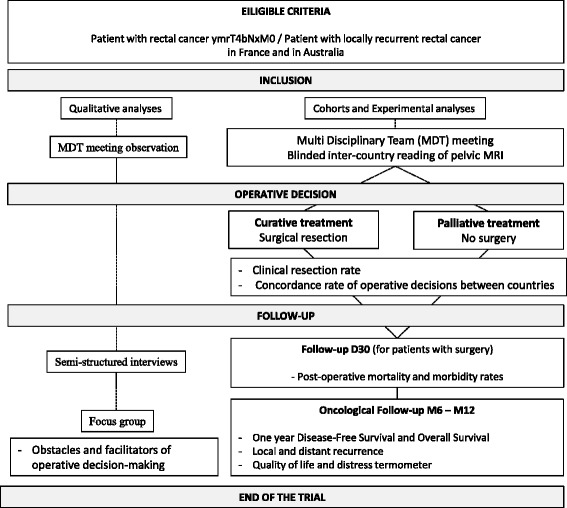
Flow chart PelviCare trial

A flowchart is given in Fig. 1.

### Endpoints

#### Primary endpoint

The primary end point of the study is the clinical resection rates in both countries. It will be expressed as a percentage and corresponding to the ratio between the number of patients operated and the number of patients discussed in colorectal MDT meetings for PRC-bTME and LRRC. These rates will be expressed separately in each country and compared.

#### Secondary endpoints

Secondary end points of the study are:

- The concordance rate of operative decisions between France and Australia. An analysis of concordance between French and Australian operative decisions will be carried out through the radiological (or theoretical) resectability rate, expressed as a percentage and corresponding, after blind inter-country reading of pelvic MRIs, to the ratio between the number of patients judged to have resectable tumours and the number of all MRI re-reading.
- The post-operative morbidity and mortality rates within 30 postoperative days for patients with curative treatment intent will be evaluated according to the Dindo classification [37].
- The oncological outcomes, based on the R0 resection rate and the 1-year overall survival and disease-free survival.
- The comparison of quality of life, according to MOS SF-36 and FACT-C questionnaires.
- The comparison of the distress level of patients according to the distress thermomether scale.

Qualitative analyses have its own end points with regards to verbal and non-verbal elements during therapeutic decisions: spatial positioning of the participants, speaking and division of speaking time, denouement of the exchanges (temporal dynamic), argument and counter-arguments, silences, overlapping of words, distribution of speaking time, leader effect, et al. identification of individual (intra-personal level), collective (inter-individual level) and contextual (institutional and socio-cultural level) obstacles and facilitators of operative decision-making.

### Statistical considerations

#### Caculation of the size of the population

The principal objective is to compare care management practices for patients suffering from PRC-bTME and LRRC, between France and Australia. The primary end point is the clinical resection rate. We make the hypothesis that in France the clinical resection rate will be 20 % and that in Australia it will be 40 %. In this situation, the calculation based on the formula of Yates’ chi-squared test with a risk α of 5 % and a power 1-β of 90 %, it must include at least 120 patients in each country, for a total of 240 patients (nQuery Advisor 7.0®).

One of the secondary end points of the study consists in the analysis of concordance between French surgical decisions and Australian ones (more specifically regarding the indication to resectability). We expect the concordance of the indication to resectability between France and Australia to be moderate. Kappa coefficient of 0.6, and show that this is different from 0.4 with a power of 80 % and an alpha risk of 5 %. The necessary number of patients to include is 240.

#### Statistical analysis

The data will be analysed by the biostatistician of the Methodology and Data Management Centre (Centre de Méthodologie et de Gestion des données – USMR). The analyses will be carried out with SAS® software (version 9.2 and later versions). The quantitative variables will be described in terms of effective, average, standard deviation and confidence interval at 95 % of the average, median, range and interquartile range. The qualitative variables will be described in terms of effective, percentage and confidence interval at 95 % according to exact binomial law. The risks of first occurrence of events will be estimated by the non-parametric method of Kaplan-Meier and compared with the Log-Rank test. A model of proportional risks from Cox will be used in order to take adjustments into account if necessary. The log-linear and risk proportionality hypotheses will be systematically verified.

For qualitative data, in each country, the interviews, focus groups and verbal interactions during MDT meetings (“real” and “theoretical”) will be submitted for thematic categorical content analyses (with the help of the NVivo9 software). This will allow for updating the principal obstacles and facilitators of operatory decisions in the framework of MDT meetings of patients with PRC-bTME and LRRC.

The concordance on the indication to the resectability between France and Australia will be estimated by the Kappa coefficient of Cohen. The confidence interval of 95 % of this estimation will be calculated by Fisher’s exact method based on distribution.

## Discussion

Despite the combination of promising oncological outcomes in the case of curative surgical resection in one hand, and the difficulties of both operative decision-making and postoperative management in the other hand, only a small proportion of patients are referred to a specialist centre in France similar to the UK [30]. Therefore, management of these patients is highly variable without agreement or consensus [1]. These variations in practice can likely be explained by health system structural differences, as well as cultural differences.

Benchmarking can be defined as a comparison and learning process that consists of observing what referent structures are doing with regards to the material and how they are doing it, with the objective of drawing information in order to improve one’s own domain of activity. Benchmarking is not limited to the comparative measure of results, but also includes the comparative analysis of processes and circuits required to change clinical practice and to allow continuous improvement of quality of care. Finally, the qualitative experiment will give potential explicative perspectives to quantitative outcomes.

In this context, the potential benefits of the PelviCare trial would be to share individual and collective mechanisms of operative decision-making as well as the whole process management of patients with PRC-bTME and LRRC in order to understand the difference of patients’ care between France and Australia. This would likely ensure equitable practice and optimal clinical pathways to improve survival outcomes and quality of life for these patients.

## Abbreviations

AJCC: American Joint Committee on Cancer
CRF: Clinical Report Form
FACT-C: Functional Assessment of Cancer Therapy-Colorectal version
LRRC: Locally Recurrent Rectal Cancer
MDT: Multidisciplinary Team
MOS SF36: Medical Outcome Study Short Form 36 item
MRI: Magnetic Resonance Imaging
PRC-bTME: Primary rectal cancer –beyond Total Mesorectal Excision plane
R0: microscopically free resection margin
R1: resection margin microscopically involved by the cancer
UK: United Kingdom
